# Consequences of advanced aging on renal function in chronic hyperandrogenemic female rat model: implications for aging women with polycystic ovary syndrome

**DOI:** 10.14814/phy2.13461

**Published:** 2017-10-19

**Authors:** Chetan N. Patil, Lorraine C. Racusen, Jane F. Reckelhoff

**Affiliations:** ^1^ Department of Physiology The Women's Health Research Center University of Mississippi Medical Center Jackson Mississippi; ^2^ Department of Biophysics The Women's Health Research Center University of Mississippi Medical Center Jackson Mississippi; ^3^ Department of Pathology Johns Hopkins University School of Medicine Baltimore Maryland

**Keywords:** Female, GFR, kidney

## Abstract

Polycystic ovary syndrome (PCOS) is the most common endocrine and reproductive disorder in premenopausal women, characterized by hyperandrogenemia, metabolic syndrome, and inflammation. Women who had PCOS during their reproductive years remain hyperandrogenemic after menopause. The consequence of chronic hyperandrogenemia with advanced aging has not been studied to our knowledge. We have characterized a model of hyperandrogenemia in female rats and have aged them to 22–25 months to mimic advanced aging in hyperandrogenemic women, and tested the hypothesis that chronic exposure to hyperandrogenemia with aging has a deleterious effect on renal function. Female rats were chronically implanted with dihydrotestosterone pellets (DHT 7.5 mg/90 days) that were changed every 85 days or placebo pellets, and renal function was measured by clearance methods. Aging DHT‐treated females had a threefold higher level of DHT with significantly higher body weight, mean arterial pressure, left kidney weight, proteinuria, and kidney injury molecule‐1 (KIM‐1), than did age‐matched controls. In addition, DHT‐treated‐old females had a 60% reduction in glomerular filtration rate, 40% reduction in renal plasma flow, and significant reduction in urinary nitrate and nitrite excretion (UNOxV), an index of nitric oxide production. Morphological examination of kidneys showed that old DHT‐treated females had significant focal segmental glomerulosclerosis, global sclerosis, and interstitial fibrosis compared to controls. Thus chronic hyperandrogenemia that persists into old age in females is associated with renal injury. These data suggest that women with chronic hyperandrogenemia such as in PCOS may be at increased risk for development of chronic kidney disease with advanced age.

## Introduction

Polycystic ovary syndrome (PCOS) is the most common endocrine and reproductive disorder, affecting 10–15% of women of all ethnicities (Azziz et al. 2006; Escobar‐Morreale and San Millan 2007; Diamanti‐Kandarakis 2008). PCOS can occur as early as menarche, and women with PCOS manifest with hirsutism, hyperandrogenemia, peripheral insulin resistance, dyslipidemia, and often have visceral adiposity. Studies in postmenopausal women who had PCOS show that the elevated androgen levels remain (Patel et al. 2008; Schmidt et al. 2011), but whether aging women who have had PCOS prior to menopause are at increased risk of cardiovascular disease later in life is controversial. In the Coronary Artery Risk Development in Young Adults (CARDIA) study, women with PCOS, defined as irregular menses and hyperandrogenemia, with a mean age 45 years, were 2.7 times more likely to develop coronary artery calcification and increased carotid intima media thickness, compared to women who had either isolated oligomennorrhea or isolated hyperandrogenemia (Calderon‐Margalit et al. 2014). However, by 62 years of age, in the Women's Ischemia Syndrome Evaluation (WISE) studies, women with clinical features of PCOS were compared with age‐matched non‐PCOS women, but who had similar characteristics, such as insulin resistance, hypertension, dyslipidemia, waist circumference and BMI. PCOS women did not show greater mortality in this 10‐year follow‐up than non‐PCOS women (Merz et al. 2016). Laughlin and colleagues reported that postmenopausal women (aged 50–91 years) in the highest two quintiles of testosterone levels had a 1.96‐fold higher risk of coronary heart disease (Laughlin et al. 2010). Thus while studies have been done to evaluate coronary disease and hypertension in older postmenopausal women with PCOS, no studies have been done to our knowledge to determine whether they have increased risk for renal disease.

In recent years, we have used a model of chronic hyperandrogenemia with implantation of dihydrotestosterone (DHT) pellets beginning at 4–5 weeks of age in female Sprague–Dawley rats (Yanes et al. 2011). By 16 weeks of age, the hyperandrogenemic females exhibit elevated blood pressure and modest proteinuria with elevated glomerular filtration rate (GFR), likely due to hyperglycemia associated with insulin resistance (Yanes et al. 2011). The rise in blood pressure and metabolic dysfunction manifested in our rat model mimics observation made in women with PCOS. We have recently characterized the effect of menopause on hyperandrogenemic females at 13–14 months of age (Dalmasso et al. 2016). At this age, the hyperandrogenemic females have frank hypertension (≥25 mmHg increase in MAP compared to placebo control) and 20% reductions in GFR with proteinuria (Dalmasso et al. 2016).

This study tested the hypothesis that chronic hyperandrogenemia leads to further renal injury and dysfunction with advanced age. The study was done in female rats that were chronically implanted with DHT beginning at 4–5 weeks of age and studied at 22–25 months of age.

## Materials and Methods

### Animals

Female Sprague–Dawley rats, aged 3 weeks, were obtained from the vendor (Harlan Sprague–Dawley, Indianapolis, IN) and allowed to equilibrate in a temperature‐controlled environment with 12:12‐h light:dark cycle for at least 1 week. Rats were then implanted with DHT (7.5 mg/90 days, *n* = 6/grp) or placebo pellets (*n* = 6/grp) beginning at 5–6 weeks of age; pellets were changed every 85 days as aging progressed (Yanes et al. 2011; Dalmasso et al. 2016). Rats were allowed ad libitum access to rat chow (Teklad #8640) and tap water, and aged to 22–25 months of age. All procedures were approved by the Animal Care and Use Committee of the University of Mississippi Medical Center and followed the *Guidelines for the Care and Use of Laboratory Animals, 2011 edition*.

### Plasma dihydrotestosterone

Plasma DHT was measured by radioimmunoassay as previously described (Yanes et al. 2011; Dalmasso et al. 2016).

### Urine analysis

Old hyperandrogenemic female and control rats were placed in metabolism cages for 1 day to equilibrate with ad libitum access to food and water, and on the second day, urine was collected for 24 h with rats fasted but given ad libitum access to water. Proteinuria was measured using the Coomassie method and a commercially available reagent (Biorad, Richmond, California), as previously described (Yanes et al. 2011; Dalmasso et al. 2016). Data are expressed as mg/24 h or factored for GFR. Urinary nitrate/nitrite excretion (UNOxV) from old placebo control and DHT rats was measured as we previously described (Reckelhoff et al. 1994; Patil et al. 2016). Kidney injury molecule‐1 (KIM‐1), proximal tubular injury biomarker, was measured in urine using an ELISA assay kit (R&D Systems, Cat# RKM 100), according to the manufacturer's instructions, as we previously described (Patil et al. 2016). KIM‐1 levels in urine were factored for the volume of urine collected over the first 24 h after reperfusion. Data are expressed as picograms excreted per 24 h.

### Renal function study

Renal function was measured by the clearance technique, as we previously described (Reckelhoff et al. 1998; Maranon et al. 2015). Briefly, old females rats, DHT or placebo treated (*n* = 6/grp) were anesthetized by intraperitoneal injection of the thiobarbiturate, Inactin (100 mg/kg body weight), and placed on a temperature‐regulated surgery table to maintain body temperature at 37 ± 0.5°C. Catheters were placed in the femoral artery (blood pressure monitoring and blood sampling) and femoral vein for infusion of artificial rat plasma (2.5 g/dL bovine immunoglobulin, 2.5 g/dL bovine serum albumin in Ringer's solution) at 12.5 mL/kg per hour for 45 min during the preparatory surgery and thereafter at 1.5 mL/kg per hour throughout the experimental period to maintain an euvolemic preparation. A catheter was placed in the second femoral vein for infusion of ^3^H‐Inulin (3 *μ*Ci/mL, 1 mL/h). A catheter was placed in the left ureter for urine sample collection. Following 1 h equilibration, two timed (15–30 min) urine samples and a midpoint arterial blood sample were collected. At the end of the experiment, a 23 g needle connected to V3 tubing was inserted into the left renal vein, and renal venous blood samples were drawn along with an arterial blood sample for calculation of renal plasma flow. At the end of the study, kidneys were removed and weighed, and placed into 10% buffered formalin. ^3^H‐inulin was measured as cpm in urine and plasma using a gamma counter, and GFR, renal plasma flow, and renal vascular resistance were calculated.

### Renal morphology study

Formalin‐fixed hemisected kidneys were embedded in paraffin and 5 *μ*m serial sections were cut and stained with Periodic acid–Schiff (PAS). Renal morphology was performed by an observer (Lorraine C. Racusen) who was blinded to the study groups.

### Statistical analyses

Data are presented as mean ± standard error of the mean and comparisons were made by *t*‐test test using Prism software with *P* < 0.05 considered significant.

## Results

### Characteristics of aging DHT‐treated and control female rats

As shown in Table 1 and as we have previously shown in this model, DHT levels were approximately threefold higher in DHT‐treated‐old females than in controls. Old DHT‐treated females had significantly higher body weight, left kidney weight, kidney weight to body weight ratios, and hematocrits than did age‐matched controls.

**Table 1:** Characteristics of aging hyperandrogenemic (DHT) female rats compared to placebo controls

	BW (g)	LKW (g)	LKW/BW	Hct (%)	DHT (pg/mL)	MAP (mmHg)	GFR (mL/min/g·KW)	RPF (mL/min/g·KW)	FF
Control (*n* = 6/grp)	309.33 ± 8.87	0.838 ± 0.021	0.0027 ± 0.0001	39 ± 2	79.26 ± 9.93	110 ± 5	1.004 ± 0.087	4.08 ± 0.56	0.25 ± 0.01
DHT (*n* = 6/grp)	420.83 ± 18.46	1.492 ± 0.113	0.0035 ± 0.0003	47 ± 2	272.98 ± 87.08	130 ± 6	0.422 ± 0.083	2.31 ± 0.45	0.18 ± 0.01
*t*‐test (*P* value)	0.0001	<0.0001	0.0343	0.0025	0.0386	0.0141	0.0003	0.0223	0.005

BW, body weight; LKW, left kidney weight; LKW/BW, Left kidney weight to body weight ratio; DHT, plasma dihydrotestosterone levels; MAP, mean arterial pressure; GFR, glomerular filtration rate; RPF, renal plasma flow; FF, filtration fraction.

### Blood pressure, renal function, and morphology

Mean arterial pressure (MAP) was increased by approximately 25 mm Hg, GFR was reduced by 60%, and renal plasma flow was reduced by approximately 40% in old DHT‐treated females compared to controls (Table 1). Filtration fraction was also lower in old DHT females than controls (Table 1). As shown in Table 2, proteinuria levels were significantly higher in old DHT‐treated females whether expressed as mg excreted per 24 h or mg excreted per 24 h per GFR. Urinary nitrate/nitrite excretion was significantly reduced, and Kidney Injury Molecule‐1 (KIM‐1) was significantly elevated in old DHT females, compare to controls (Table 2).

**Table 2:** Urinary protein, nitrate/nitrite, and KIM‐1 excretion in aging hyperandrogenemic (DHT) female rats compared to placebo controls

	UPrV (mg/24 h)	UPrV (mg/24 h)per GFR	UNOxV (*μ*mol/24 h/kg BW)	KIM‐1 (pg/24 h)
Control (*n *= 6/grp)	4.98 ± 0.83	5.095 ± 0.92	2.00 ± 0.30	5907 ± 1147
DHT (*n* = 6/grp)	37.21 ± 12.92	102.5 ± 39.26	0.88 ± 0.34	12368 ± 2038
*t*‐test (*P* value)	0.0321	0.0325	0.0365	<0.0439

UPrV, urinary protein excretion; UNOxV, urinary nitrate/nitrite excretion; KIM‐1, kidney injury molecule‐1 excretion.

**Figure 1:**
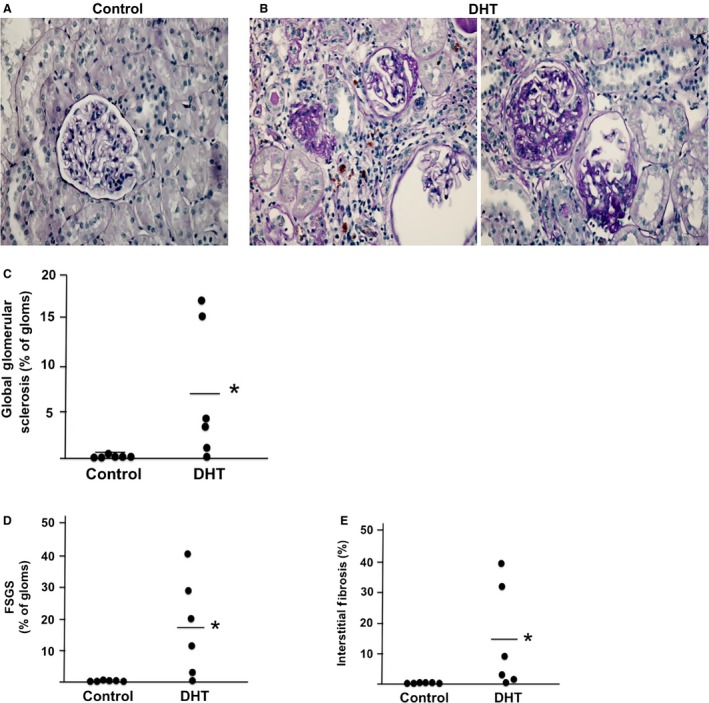
Morphological analyses of kidneys from aging hyperandrogenemic females and controls. (A and B) Representative micrograph of kidneys of control (A) and DHT‐treated (B) aging female kidneys (magnification = 20×). Evaluation of PAS‐stained sections was performed for (C) Global sclerosis; (D) Focal segmental glomerulosclerosis (FSGS); (E) Interstitial fibrosis (*n* = 6/group for all figures). *P < 0.5, compared with control group.

As shown in Figure 1, morphological evaluation showed that kidneys from old DHT‐treated females had greater global sclerosis, focal segmental glomerular sclerosis (FSGS), and interstitial fibrosis compared to age‐matched control females that had little global sclerosis, tubular atrophy or interstitial fibrosis.

## Discussion

In this study, we found that chronic hyperandrogenemia in females from 4 to 5 weeks of age to 22–25 months of age results in 60% reductions in GFR, 40% reductions in renal plasma flow, significant increases in proteinuria and KIM‐1, and reductions in UNOxV, compared to age‐matched control females. The data suggest that with aging, chronic hyperandrogenemia produces chronic kidney disease in these female rats.

In young women, the phenotype of PCOS has been well characterized by many groups from different nationalities (Azziz et al. 2006; Escobar‐Morreale and San Millan 2007; Diamanti‐Kandarakis 2008). The diagnosis is made with two of the three characteristics being present: polycystic ovaries, hyperandrogenemia, and menstrual abnormalities or infertility (Azziz et al. 2006). Women with PCOS typically have insulin resistance with or without obesity although more women in industrialized countries typically exhibit obesity than in third world countries (Azziz et al. 2006; Escobar‐Morreale and San Millan 2007; Diamanti‐Kandarakis 2008). They also exhibit hyperlipidemia, increased inflammation, and symptoms of elevated androgens, such as hirsutism, depending on the levels of circulating androgens. In addition, women with PCOS often exhibit elevated blood pressure compared to their age‐matched counterparts. With menopause, the elevated levels of androgens persist compared to postmenopausal women who did not suffer from PCOS during their reproductive lives, and by this time, PCOS women are typically frankly hypertensive and receiving appropriate antihypertensive therapy (Laughlin et al. 2010; Schmidt et al. 2011; Pinola et al. 2015; Merz et al. 2016).

We have characterized a model of hyperandrogenemia in female rats that has many of the same characteristics as women with PCOS (Yanes et al. 2011), and evaluated the mechanisms responsible for their elevated blood pressure. When young, the hyperandrogenemic female rats have threefold higher dihydrotestosterone levels, similar to the increase in androgens found in women with PCOS (Pinola et al. 2015), but have similar levels of estradiol and testosterone as controls with 6 day estrous cycles rather than 4 day cycles (Yanes et al. 2011), suggesting that the endogenous production of estradiol and testosterone is not downregulated by the dihydrotestosterone treatment. In addition, young hyperandrogenemic female rats exhibit obesity, insulin resistance, increases in cholesterol and inflammatory cytokines (Yanes et al. 2011), as are often found in women with PCOS. By 16 weeks of age, they exhibit modest proteinuria with elevated GFR compared to controls (Yanes et al. 2011). Young hyperandrogenemic female rats also exhibit approximately 10 mmHg increase in blood pressure (Yanes et al. 2011). The mechanisms responsible for the elevated blood pressure include activation of the sympathetic nervous system, including a role for the renal nerves (Maranon et al. 2015), just as in women (Schlaich et al. 2011). Furthermore, activation of the melanocortin 4 receptor (MC4R) contributes to the elevated blood pressure since blockade of the MC4R reduces blood pressure in the DHT‐treated females, but not controls (Maranon et al. 2015).

We recently characterized the effect of chronic hyperandrogenemia on metabolic and kidney function after estrous cycling has ceased at 12–13 months of age in our female rat model. Compared to this study of the long‐term aging effects of hyperandrogenemia, just following menopause, hyperandrogenemic female rats are hypertensive, and have proteinuria with modest (20%) reductions in GFR (Dalmasso et al. 2016). In this study then, advanced aging to 22–25 months is associated with further increases in body weight, that does not occur in age‐matched placebo controls (early postmenopausal (EPM) placebo): 307.9 ± 20.1 g (Diamanti‐Kandarakis 2008); aging placebo 22–25 months old (old): 309.33 ± 8.87 g; DHT EPM: 397.3 ± 9.4 g (Diamanti‐Kandarakis 2008); DHT old: 420.83 ± 18.46 g, *P* < 0.05, DHT old compared with DHT EPM).

As mentioned above, GFR was reduced by 20% in early postmenopausal DHT rats (Dalmasso et al. 2016), but GFR in old DHT females in this study was reduced by 60% and renal plasma flow was reduced by 40%. Filtration fraction was also reduced suggesting an increase in preclomerular resistance. Renal injury is also evident in the morphological evaluation, the proteinuria, and the KIM‐1 excretion. UNOxV is reduced in old DHT females by 60% compared to old placebo controls. In other aging models, Baylis reported that the reduction in renal plasma flow is associated with increases in the NO synthase inhibitor, ADMA, in the kidney (Baylis 2012). Future studies will be necessary to determine if ADMA levels are increased with aging in the old DHT females.

In young women with PCOS, there are little data regarding renal function. Gozukara and colleagues reported that GFR was increased in young women with PCOS from Turkey (Gozurka et al. 2015), just as we found in our young hyperandrogenemic female rats (Yanes et al. 2011). Ziaee and colleagues reported that in Iranian women, microalbuminuria was found in 53% of young women with PCOS, and was associated with insulin resistance, elevated blood pressure, and greater waist circumference (Ziaee et al. 2012), although neither GFR nor serum creatinine was measured. Caglar and colleagues and Patel and colleagues made similar findings in women with PCOS in a Rotterdam hospital study (Patel et al. 2008; Caglar et al. 2011). As noted, these studies were performed in women in their mid to early 20s. Furthermore, in all these studies the women with PCOS who exhibited microalbuminuria were hypertensive (Patel et al. 2008; Calderon‐Margalit et al. 2014), thus the elevated blood pressure may have contributed to the proteinuria.

To our knowledge, there are no studies in which GFR or proteinuria has been reported in postmenopausal women with PCOS. Often whether a woman had PCOS when she was younger is not considered to be relevant to her postmenopausal healthcare, especially if she is very elderly. In addition, sex steroid hormone levels are rarely measured, particularly if several years have passed since the menopause transition. If women with PCOS have underlying renal disease as they age, then any cardiovascular/renal event that would seem minor to normally aging women could predispose them to acute or chronic renal disease, leading to end‐stage renal disease sooner than in the general population of aging women. This is the “second hit” hypothesis. Future studies are necessary to determine if in fact there is a higher incidence of chronic renal disease in aging, postmenopausal women who had PCOS during their reproductive years. Furthermore, physicians who care for elderly women should be cognizant of whether they had PCOS when young and carefully evaluate their renal function.

## Conflict of Interest

None declared.

## References

[phy213461-bib-0001] Azziz, R. , E. Carmina , D. Dewailly , E. Diamanti‐Kandarakis , H. F. Escobar‐Morreale , W. Futterweit , et al. 2006 Positions statement: criteria for defining polycystic ovary syndrome as a predominantly hyperandrogenic syndrome: an Androgen Excess Society guideline. J. Clin. Endocrinol. Metab. 91:4237–4245. 16940456 10.1210/jc.2006-0178

[phy213461-bib-0002] Baylis, C. 2012 Sexual dimorphism: the aging kidney, involvement of nitric oxide deficiency, and angiotensin II overactivity. J. Gerontol. A Biol. Sci. Med. Sci. 67:1365–1372. 22960474 10.1093/gerona/gls171 PMC3708515

[phy213461-bib-0003] Caglar, G. S. , E. Oztas , D. Karadag , R. Pabuccu , and A. A. Eren . 2011 The association of urinary albumin excretion and metabolic complications in polycystic ovary syndrome. Eur. J. Obstet. Gynecol. Reprod. Biol. 154:57–61. 20888116 10.1016/j.ejogrb.2010.08.024

[phy213461-bib-0004] Calderon‐Margalit, R. , D. Siscovick , S. S. Merkin , E. Wang , M. L. Daviglus , P. J. Schreiner , et al. 2014 Prospective association of polycystic ovary syndrome with coronary artery calcification and carotid‐intima‐media thickness: the Coronary Artery Risk Development in Young Adults Women's study. Arterioscler. Thromb. Vasc. Biol. 34:2688–2694. 25359859 10.1161/ATVBAHA.114.304136

[phy213461-bib-0005] Dalmasso, C. , R. Maranon , C. Patil , E. Bui , M. Moulana , H. Zhang , et al. 2016 Cardiometabolic effects of chronic hyperandrogenemia in a new model of postmenopausal polycystic ovary syndrome. Endocrinology 157:2920–2927. 27145003 10.1210/en.2015-1617 PMC4929551

[phy213461-bib-0006] Diamanti‐Kandarakis, E. 2008 Polycystic ovarian syndrome: pathophysiology, molecular aspects and clinical implications. Expert Rev. Mol. Med. 10:e3. 18230193 10.1017/S1462399408000598

[phy213461-bib-0007] Escobar‐Morreale, H. F. , and J. L. San Millan . 2007 Abdominal adiposity and the polycystic ovary syndrome. Trends Endocrinol. Metab. 18:266–272. 17693095 10.1016/j.tem.2007.07.003

[phy213461-bib-0008] Gozukara, I. O. , K. H. Gozukara , S. K. Kucur , E. U. Karakilic , H. Keskin , D. Akdeniz , et al. 2015 Association of glomerular filtration rate with inflammation in polycystic ovary syndrome. Int. J. Fertil. Steril. 9:176–182. 26246875 10.22074/ijfs.2015.4238 PMC4518485

[phy213461-bib-0009] Laughlin, G. A. , V. Goodell , and E. Barrett‐Connor . 2010 Extremes of endogenous testosterone are associated with increased risk of incident coronary events in older women. J. Clin. Endocrinol. Metab. 95:740–747. 19934360 10.1210/jc.2009-1693 PMC2840853

[phy213461-bib-0010] Maranon, R. , R. Lima , F. T. Spradley , J. M. do Carmo , H. Zhang , A. D. Smith , et al. 2015 Roles for the sympathetic nervous system, renal nerves, and CNS melanocortin‐4 receptor in the elevated blood pressure in hyperandrogenemic female rats. Am. J. Physiol. Regul. Integr. Comp. Physiol. 308:R708–R713. 25695289 10.1152/ajpregu.00411.2014 PMC4398855

[phy213461-bib-0011] Merz, C. N. , L. J. Shaw , R. Azziz , F. Z. Stanczyk , G. Sopko , G. D. Braunstein , et al. 2016 Cardiovascular disease and 10‐year mortality in postmenopausal women with clinical features of polycystic ovary syndrome. J. Women's Health 25:875–881. 10.1089/jwh.2015.5441 PMC5311460 27267867

[phy213461-bib-0012] Patel, A. A. , Z. T. Bloomgarden , and W. Futterweit . 2008 Premicroalbuminuria in women with polycystic ovary syndrome: a metabolic risk marker. Endocr. Pract. 14:193–200. 18308657 10.4158/EP.14.2.193

[phy213461-bib-0013] Patil, C. N. , K. Wallace , B. D. LaMarca , M. Moulana , A. Lopez‐Ruiz , A. Soljancic , et al. 2016 Low‐dose testosterone protects against renal ischemia‐reperfusion injury by increasing renal IL‐10‐to‐TNF‐alpha ratio and attenuating T‐cell infiltration. Am. J. Physiol. Renal Physiol. 311:F395–F403. 27252490 10.1152/ajprenal.00454.2015 PMC5008676

[phy213461-bib-0014] Pinola, P. , T. T. Piltonen , J. Puurunen , E. Vanky , I. Sundstrom‐Poromaa , E. Stener‐Victorin , et al. 2015 Androgen profile through life in women with polycystic ovary syndrome: a Nordic multicenter collaboration study. J. Clin. Endocrinol. Metab. 100:3400–3407. 26192874 10.1210/jc.2015-2123

[phy213461-bib-0015] Reckelhoff, J. F. , J. A. Kellum , E. J. Blanchard , E. E. Bacon , A. J. Wesley , and W. C. Kruckeberg . 1994 Changes in nitric oxide precursor, L‐arginine, and metabolites, nitrate and nitrite, with aging. Life Sci. 55:1895–1902. 7990649 10.1016/0024-3205(94)00521-4

[phy213461-bib-0016] Reckelhoff, J. F. , H. Zhang , and J. P. Granger . 1998 Testosterone exacerbates hypertension and reduces pressure‐natriuresis in male spontaneously hypertensive rats. Hypertension 31:435–439. 9453341 10.1161/01.hyp.31.1.435

[phy213461-bib-0017] Schlaich, M. P. , N. Straznicky , M. Grima , C. Ika‐Sari , T. Dawood , F. Mahfoud , et al. 2011 Renal denervation: a potential new treatment modality for polycystic ovary syndrome? J. Hypertens. 29:991–996. 21358414 10.1097/HJH.0b013e328344db3a

[phy213461-bib-0018] Schmidt, J. , K. Landin‐Wilhelmsen , M. Brannstrom , and E. Dahlgren . 2011 Cardiovascular disease and risk factors in PCOS women of postmenopausal age: a 21‐year controlled follow‐up study. J. Clin. Endocrinol. Metab. 96:3794–3803. 21956415 10.1210/jc.2011-1677

[phy213461-bib-0019] Yanes, L. L. , D. G. Romero , M. Moulana , R. Lima , D. D. Davis , H. Zhang , et al. 2011 Cardiovascular‐renal and metabolic characterization of a rat model of polycystic ovary syndrome. Gend. Med. 8:103–115. 21536229 10.1016/j.genm.2010.11.013 PMC3093922

[phy213461-bib-0020] Ziaee, A. , S. Oveisi , A. Ghorbani , S. Hashemipour , and M. Mirenayat . 2012 Association between metabolic syndrome and premicroalbuminuria among Iranian women with Polycystic Ovary Syndrome: a case control study. Glob. J. Health Sci. 5:187–192. 23283052 10.5539/gjhs.v5n1p187 PMC4776989

